# Watson transform in quantum scattering

**DOI:** 10.1038/s41598-025-19443-3

**Published:** 2025-10-24

**Authors:** Constantinos Valagiannopoulos, Vassilios Kovanis

**Affiliations:** 1 School of Electrical & Computer Engineering, National Technical University of Athens, Athens, 15772 Greece; 2 National Security Institute, Arlington, VA 22203 USA

**Keywords:** Matter waves and particle beams, Applied mathematics

## Abstract

The scattering of high-energy quantum particles by nanoinclusions into crystalline lattices is studied. Since the typical size of the grid impurity is much larger compared to the wavelength of the produced matter waves, the canonical solutions for the wave functions given as series of spatial harmonics, converge very poorly. Therefore, Watson transform is employed to provide equivalent series that involve complex-ordered Hankel functions and possess a hugely better convergence rate. In this way, the use of a versatile tool is demonstrated allowing for rigorously solving and understanding particle interactions that occur within various research domains: from quantum emission and interference to molecular fluctuations and quantum signal processing.

## Introduction

Quantum interactions of particles with matter have been behind scientific breakthroughs across directions like accurate sensing that led to the development of superconducting quantum interference devices^1^ or long-distance quantum communication promising absolutely secure transmission^2,3^. The same quantum principles keep delivering trailblazing concepts like achieving quantum computational advantage in bosonic sampling using photons^4^ and developing qubit connectivity towards hardware able to tame intractable so far complexity^5^. Moreover, the field of quantum machine learning emerges by focusing on how to devise and implement quantum software that could generate artificial intelligence enormously faster than that of classical computers^6^. All this unprecedented research progress involving quantum interactions has stimulated long-term visions about the materials necessary in the related applications^7^ and impressive funding initiatives^8,9^ able to fuel the development of the respective technologies.

The emission of quantum particles forming matter waves lies at the heart of quantum optics^10^ while their scattering by crystalline inclusions can end up in the coupling of quantum dots with structures like nanoantennas^11^ or nanocavities^12^. Indeed, the investigation of the collisions between non-relativistic particles^13^ have led to quantum scattering interferometers^14^ that enable direct and precise measurements^15^ of ultracold atom–atom interactions. Interestingly, quantum scattering calculations are used towards understanding bimolecular reactions in the gas phase while providing detailed predictions on the dynamics and kinetics of the respective chemical processes^16^. Finally, several studies have been executed on the evaluation of quantum scattering with use of partial wave formalism^17^, spectral projections^18^ and application of optical theorem^19^.

However, most methods for quantum scattering calculation face their limits and fail once the size of the scatterer gets much larger compared to the wavelength of the impinging matter wave. Such a regime is very common since high-energy particles possess wavelengths equal to a few nanometers, while the size of typical inclusions in crystalline lattice can be hundreds of nanometers or even bigger. In electromagnetic scattering, similar convergence issues emerge and are treated via implementing the so-called Watson transform^20^ on the canonical solution series. With such an approach, the summation index ceases to be an integer while the terms of the sum are reproduced as residues of a suitable integral across a carefully selected path on the complex index plane^21^. Several modifications of the Watson transform have been performed to reduce the complexity of the calculations^22^ and offer a more clear physical interpretation of the solutions^22^. Finally, it is stressed that this method, contrary to other high-frequency approximate counterparts like geometrical optics or ray tracing^23^, is a rigorous one that has been expanded to cover elastic^24^ or acoustic^25^ interactions between waves and obstacles.

In this work, we apply the Watson transform for the quantum scattering of high-energy matter waves, emitted omni-directionally from a line source, by an impenetrable cylindrical nanoinclusion. The slow convergence of the partial waves series is demonstrated and the equivalent Watson series is obtained by involving integrals on the complex plane of the order of Hankel functions. The residues are numerically evaluated and the direct computation of the new sum is hugely accelerated compared to the canonical solution. The influence of the size of the scatterer, the location of the source and the observation point on the convergence rate, is identified. The derived results establish Watson transform as a versatile tool to study quantum scattering. It can be done by determining rigorously the wave functions in setups hosting further quantum interactions such as electron interference, quantum signal processing and atomic or molecular fluctuations.

**Fig. 1:**
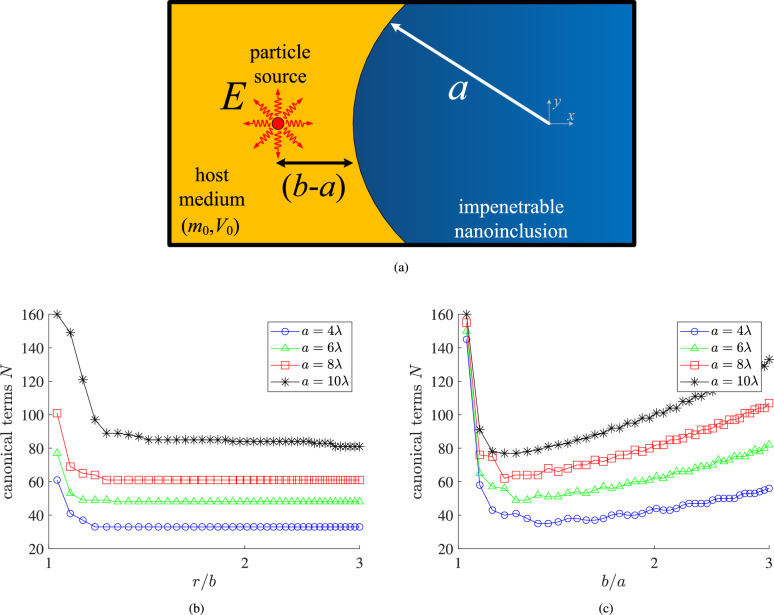
(**a**) The investigated setup: an omni-directional source of quantum particles produces cylindrical matter waves of high energy *E* (corresponding to wavelength $\lambda$) that are scattered by a cylindrical impenetrable nanoinclusion of radius *a*. The source is positioned at distance $(b-a)$ from the surface of the scatterer and is embedded into a medium of potential $V_0$ and effective mass $m_0$. (**b**) The number of terms *N* that are needed for the canonical solution to converge as a function of the polar distance *r* normalized by the source location for various sizes *a* in terms of $\lambda$ ($\varphi =\pi /4$). (**c**) The number of terms *N* that are needed for the canonical solution to converge as a function of the radii ratio *b*/*a* for various sizes *a* in terms of $\lambda$ ($r=g \equiv (a+b)/2$ and $\varphi =\pi /4$).

## Canonical series: slow convergence

In Fig. 1a we observe a lengthy cylindrical inclusion of radius *a* embedded into a medium characterized by an effective electron mass $m_0$ and a potential level $V_0$ which can be taken equal to zero for simplicity. The structure is excited by an omni-directional source of quantum particles of energy *E* positioned at distance $(b-a)$ from the cylinder’s surface. Importantly, the inclusion is impenetrable calling for vanishing wave function ($\Psi =0$) across its entire volume while the quantum particles form cylindrical matter waves of wavenumber $k_0\equiv 2\pi /\lambda =\sqrt{2 m_0 E}/\hbar$. It should be remarked that we do not take into account the impurity of phonon scattering; as a result, the cylindrical electron wave is regarded as coherent; hence, the impinging particle beam can be represented by a specific wave vector ${\mathbf {k}}=k_0\hat{{\mathbf {r}}}$. The structure is practically invariant along the cylinder’s axis and, thus, the problem is two-dimensional involving the Cartesian (*x*, *y*) or the polar $(r,\varphi )$ coordinates, with $-\pi<\varphi <\pi$, interchangeably. It should be stressed that our approach is semi-classical in the sense that the background medium and the impenetrable inclusion are treated classically, while only the source produces particles with quantum features. In fact, the host’s potential $V_0$ expresses the energy required to extract an electron from its volume to free space. In the same semi-classical framework, the effective mass $m_0$ takes into account the bands formed due to the crystalline lattice of the background medium and, accordingly, the trajectory of an electron therein is emulated by that of a free-moving particle of different mass ($m_0$).

By solving the time-independent Schrödinger’s equation and imposing the necessary boundary conditions^26,27^, the wave function takes one form when the observation point $(r,\varphi )$ lies between the source and the nanoinclusion ($a<r<b$, internal, $\Psi _\textrm{int}$) and another when it is placed outside ($r>b$, external, $\Psi _\textrm{ext}$). The respective expressions are equal to:

1 $$\begin{aligned} \Psi (r,\varphi )=\left\{ \begin{array}{cc} \frac{f_\textrm{int}(0)}{2}+\sum _{n=1}^{+\infty }f_\textrm{int}(n) & ,a<r<b \\ \frac{f_\textrm{ext}(0)}{2}+\sum _{n=1}^{+\infty }f_\textrm{ext}(n) & ,r>b \end{array}\right. , \end{aligned}$$

where the functions $f_\mathrm{int/ext}(n)\equiv f(n)$ are given by:

2a $$\begin{aligned} f_\textrm{int}(n)=\frac{H_{n}^{(1)}(k_0 b)}{H_{n}^{(1)}(k_0 a)} (-1)^n \cos (n\varphi ) \left[ H_{n}^{(1)}(k_0 a)H_{n}^{(2)}(k_0 r)-H_{n}^{(1)}(k_0 r)H_{n}^{(2)}(k_0 a)\right] , \end{aligned}$$

2b $$\begin{aligned} f_\textrm{ext}(n)=\frac{H_{n}^{(1)}(k_0 r)}{H_{n}^{(1)}(k_0 a)} (-1)^n \cos (n\varphi ) \left[ H_{n}^{(1)}(k_0 a)H_{n}^{(2)}(k_0 b)-H_{n}^{(1)}(k_0 b)H_{n}^{(2)}(k_0 a)\right] , \end{aligned}$$

and $H_{n}^{(1)/(2)}(z)$ are the Hankel functions of 1-st or 2-nd type, order *n* and argument *z*.

The general terms *f*(*n*) of (2) behave for $n\rightarrow +\infty$ as follows^28^:

3 $$\begin{aligned} f(n)\sim \frac{2{\textrm{i}}}{\pi }(-1)^n\frac{\cos (n\varphi )}{n} \frac{2+{\textrm{i}}\left( \frac{e k_0 \max (r, b)}{2 n}\right) ^{2n}}{2+{\textrm{i}}\left( \frac{e k_0 a}{2 n}\right) ^{2n}} \left[ \left( \frac{a^2}{rb}\right) ^n-\left( \frac{\min (r,b)}{\max (r,b)}\right) ^n\right] . \end{aligned}$$

Obviously, the series in (1) are convergent but, as one can understand from (3), the convergence is slower the bigger the quantity $a/\lambda$ gets. The wavelength $\lambda$, especially for high-energy matter waves, will be about one nanometer while the inclusion will be micrometer-sized; therefore, the convergence of canonical solutions (1) is expected to be particularly slow for real-world configurations. It is noted that, given the aforementioned semi-classical approach, we treat the formulated boundary value problem in complete analogy with electromagnetics and we do not make additional amendments for the behavior of the scatterer and the source in the quantum scale. Specifically, we assume that the quantum canonicity is not broken and the atomic crystal of the host material gets perfectly zipped with the respective lattice of the nanoinclusion, around their common boundary.

In Fig. 1b, we represent the necessary number *N* of terms in order to achieve convergence for the canonical formula $\Psi _\textrm{ext}(r,\varphi )$ of (1), as function of the radial distance *r* normalized by the source location *b*, for various optical sizes of the nanoinclusion $a/\lambda$. It is clear that the more distant the observation point gets, the easier the numerical evaluation of the canonical solution becomes since the bases $a^2/(rb)$ and *b*/*r* in the exponential terms of (3), decrease. In addition, as anticipated, the convergence is harder for optically larger inclusions due to the presence of the term $(k_0a)^{2n}$ in the denominator of (3).

In Fig. 1c, we show the number of terms *N* guaranteeing convergence for the expression $\Psi _\textrm{int}(r,\varphi )$ of (1) at $r=g \equiv (a+b)/2$, as function of the radii ratio *b*/*a* for several optical sizes of the inclusion $a/\lambda$. Naturally, when $b\rightarrow a$, the source is located very close to the cylindrical surface and the observation point is also forced to touch the inclusion at which $\Psi _\textrm{int}=0$, which renders the convergence challenging. Note that *N* drops rapidly when the quantum emitter gets a bit more distant from the surface. However, as *b*/*a* increases, the convergence becomes harder because of the term $(k_0b)^{2n}$ in the numerator of (1). As far as the influence of the size 2*a* of the scatterer normalized by the operational wavelength $\lambda$ is concerned, the term $(k_0a)^{2n}$ in the denominator of (3) calls again for more terms *N*.

**Fig. 2:**
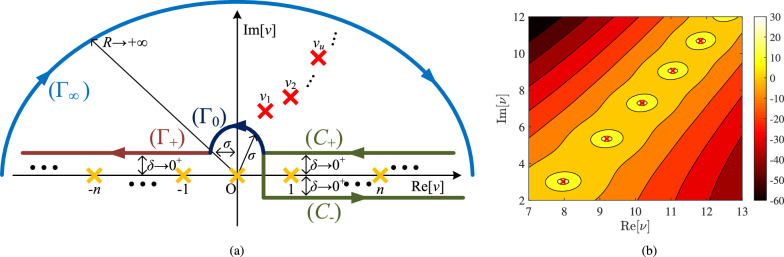
(**a**) Various integration paths across the complex order plane $({\textrm{Re}}[\nu ],{\textrm{Im}}[\nu ])$ of Hankel function. The green line $(C_+)\cup (C_-)$ gives as residues the terms of the canonical solution. Due to the odd integrand function, the integral along the path $(C_-)$ equals to that along the brown path $(\Gamma _+)$ while the gap between $(C_+)$ and $(\Gamma _+)$ is bridged by a dark blue semi-circle of radius $\sigma$. The integral dies exponentially at the infinite light blue semi-circle $(\Gamma _{\infty })$ and, thus, the integral is computed as the sum of residues corresponding to the complex zeros $\nu _u$ for $u\in {\mathbb {N}}^*$ of $H^{(1)}_{\nu }(k_0a)$. (**b**) The quantity $1/|H^{(1)}_{\nu }(k_0a)|$ in decibels for $k_0a=2\pi$. Red crosses mark the poles of the function.

## Watson series: fast convergence

It should be stressed that in actual quantum interactions we have $k_0a\gg 1$; as a result, the number of required canonical terms *N* to evaluate both the series in (1) is much larger than these indicated in Figs. 1b,c. Therefore, the direct evaluation of $\Psi (r,\varphi )$ once $a\gg \lambda$, is a quite demanding task. To remedy such an issue, we will employ Watson transform^20^. The sum of a general term *f*(*n*) is replaced by an integral along a suitable path on the complex $\nu ={\textrm{Re}}[\nu ]+{\textrm{i}}~{\textrm{Im}}[\nu ]$ plane of a properly defined integrand function $F_\mathrm{int/ext}(\nu )\equiv F(\nu )$. In particular, our selected functions are shown below:

4a $$\begin{aligned} F_\textrm{int}(\nu )=\frac{1}{2 \pi {\textrm{i}}}\frac{H_{\nu }^{(1)}(k_0 b)}{H_{\nu }^{(1)}(k_0 a)} \frac{\pi }{\sin (\nu \pi )} \cos (\nu \varphi ) \left[ H_{\nu }^{(1)}(k_0 a)H_{\nu }^{(2)}(k_0 r)-H_{\nu }^{(1)}(k_0 r)H_{\nu }^{(2)}(k_0 a)\right] , \end{aligned}$$

4b $$\begin{aligned} F_\textrm{ext}(\nu )=\frac{1}{2 \pi {\textrm{i}}}\frac{H_{\nu }^{(1)}(k_0 r)}{H_{\nu }^{(1)}(k_0 a)} \frac{\pi }{\sin (\nu \pi )} \cos (\nu \varphi ) \left[ H_{\nu }^{(1)}(k_0 a)H_{\nu }^{(2)}(k_0 b)-H_{\nu }^{(1)}(k_0 b)H_{\nu }^{(2)}(k_0 a)\right] , \end{aligned}$$

while the integration path comprises two green lines $(C_{\pm })$ infinitesimally ($\delta \rightarrow 0^+$) above and below the real positive semi-axis respectively, as shown in Fig. 2a. Indeed, according to residue theorem, the poles of (4) are at the integers $\nu =n\in {\mathbb {N}}$ excluding the term $\nu =n=0$ since the leftmost point of the path $(C_{+})\cup (C_{-})$ has a distance $\sigma$ from the imaginary axis ${\textrm{Re}}[\nu ]=0$. Notice that the terms $(-1)^n$ in (2) are produced as the residues of the factors $\pi /\sin (\nu \pi )$ in (4).

Given the fact that the functions (4) are odd ($F(-\nu )=-F(\nu )$), the integral of $F(\nu )$ along $(C_{-})$ equals to that along $(\Gamma _{+})$ brown path, we obtain:

5 $$\begin{aligned} \sum _{n=1}^{+\infty }f(n)=\int _{(C_{+})\cup (C_{-})}F(\nu ){\textrm{d}}\nu =\int _{(C_{+})\cup (\Gamma _0) \cup (\Gamma _{+})}F(\nu ){\textrm{d}}\nu -\frac{f(0)}{2}. \end{aligned}$$

The equality $\int _{(\Gamma _0)}F(\nu ){\textrm{d}}\nu =-f(0)/2$ has been used, which is derived based on the odd property of $F(\nu )$ combined with the residue theorem for $\sigma \rightarrow 0^{+}$ and a circular path around the origin with radius $\sigma$. With use of expansions of Hankel functions^29,30^ for large complex orders, the behavior of $F(\nu )$ across the light blue path $(\Gamma _{\infty })$, at which $\nu \rightarrow \infty$, is described by:

6 $$\begin{aligned} |F(\nu )|\sim \frac{e^{-{\textrm{Im}}[\nu ] (\pi -\varphi )}}{\pi |\nu |} \times \left[ \left( \frac{a^2}{rb}\right) ^{|{\textrm{Re}}[\nu ]|}+\left( \frac{\min (r,b)}{\max (r,b)}\right) ^{|{\textrm{Re}}[\nu ]|}\right] , \end{aligned}$$

for $-\pi<\varphi <\pi$. Hence, the integrand function vanishes exponentially along $(\Gamma _{\infty })$, where ${\textrm{Im}}[\nu ]>0$; as a result, the respective integral at the semi-circle with radius $R\rightarrow +\infty$ does not contribute to the final outcome: $\int _{(C_{\infty })}F(\nu ){\textrm{d}}\nu =0$.

In this way, we may write:

7 $$\begin{aligned} (1) {\mathop{\Longrightarrow}^{(5)}} \Psi(r,\varphi)=\int_{(C_{+})\cup (\Gamma_0) \cup (\Gamma_{+})\cup (C_{\infty})}F(\nu){\rm d}\nu \Rightarrow \Psi(r,\varphi)=-2\pi {\rm i} \sum_{u=1}^{+\infty}{\rm Res}[F(\nu), \nu=\nu_u],\end{aligned}$$

where $\nu _u$ for $u\in {\mathbb {N}}$ are the (simple) poles of $F(\nu )$ across the upper complex plane ${\textrm{Im}}[\nu ]>0$, namely, the solutions of the transcendental equation $H_{\nu }^{(1)}(k_0a)=0$, since all the other functions involved in (4) are analytic. They are indicated by red crosses in the sketch of Fig. 2a. In addition, in Fig. 2b and with use of Wolfram Mathematica ®, we sketch^31^ the variation of $1/|H_{\nu }^{(1)}(k_0a)|$ in decibels across the complex plane $({\textrm{Re}}[\nu ],{\textrm{Im}}[\nu ])$ of order $\nu$, for $k_0a=2\pi$. Similarly, we indicate the five first poles of the function with red markers $\times$. One directly observes that for increasing *u,* both the real and imaginary parts of $\nu _u$ get bigger.

**Fig. 3:**
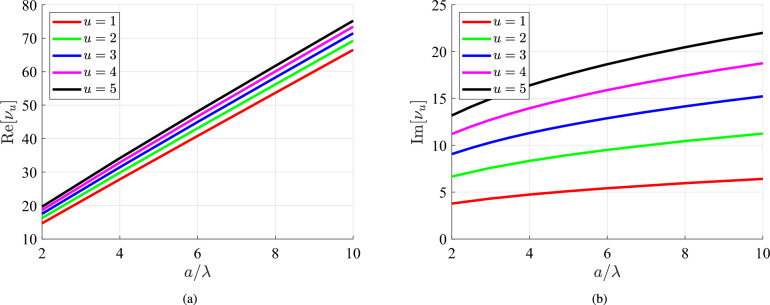
The zeros $\nu =\nu _u$ with $u\in {\mathbb {N}}^*$ of the equation $H_{\nu }^{(1)}(k_0a)=0$ as functions of the size of the nanoiclusion *a* divided by the wavelength $\lambda$ of the impinging matter wave for several sequence numbers *u*. (a) Real parts ${\textrm{Re}}[\nu _u]$ of complex roots. (b) Imaginary parts ${\textrm{Im}}[\nu _u]$ of complex roots.

In Fig. 3a, we represent the real part of these poles ${\textrm{Re}}[\nu _u]$ with respect to the size of the scatterer divided by the wavelength $\lambda$ of the matter waves, for $u=1,\cdots , 5$. As also can be understood from Fig. 2b, the real parts of $\nu _u$ are positive while they are linearly increasing with $a/\lambda$. In Fig. 3b, we show the imaginary part of the poles ${\textrm{Im}}[\nu _u]$ and we notice the upward sloping trends. Needless to say that ${\textrm{Im}}[\nu _u]$ are smaller compared to ${\textrm{Re}}[\nu _u]$, as it becomes apparent by inspection of Fig. 2b. It is important to remark that Fig. 3 provides a useful index for a nontrivial process: the exact determination of roots across the complex plane $\nu$ of the order of Hankel/Bessel functions. Indeed, many extensively used computing platforms are not able to evaluate accurately the quantities $H_{\nu }^{(1)/(2)}(z)$ for $\nu \in {\mathbb {C}}$, let alone solve, with respect to $\nu \in {\mathbb {C}}$, transcendental equations involving these functions. When it comes to the residues of (7) that require computation of the derivatives with respect to $\nu$, they are done numerically, again via specialized software. In particular, the residues of (7) are determined by evaluating (4) at the poles $\nu =\nu _u$ after replacing $H_{\nu }^{(1)}(k_0 a)$ in the respective denominators by their derivatives with respect to complex order: $\partial H_{\nu }^{(1)}(k_0 a)/\partial \nu$.

**Fig. 4:**
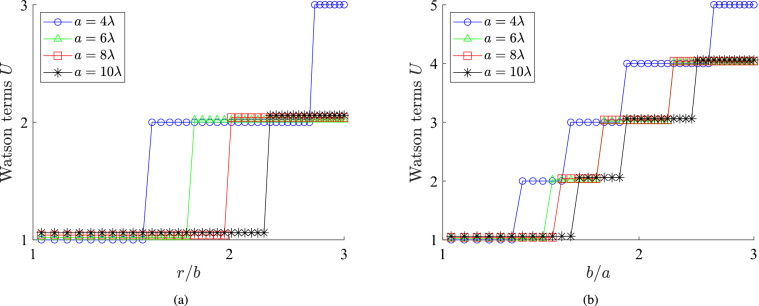
(**a**) The number of terms *U* that are needed for the Watson series to converge as a function of the polar distance *r* normalized by the source for various sizes *a* in terms of $\lambda$ ($\varphi =\pi /4$). (**b**) The number of terms *U* that are needed for the Watson series to converge as a function of the radii ratio *b*/*a* for various sizes *a* in terms of $\lambda$ ($r=g \equiv (a+b)/2$ and $\varphi =\pi /4$).

To demonstrate the fast convergence of the Watson series, we should find the necessary number of terms *U* for successful evaluation of (7) and compare with the respective numbers *N* of Figs. 1b,c, that refer to series (1). In Fig. 4a, we consider exactly the same cases as in Fig. 1b and represent the number *U* that $\Psi _\textrm{ext}$ demands for convergence when computed via (7). One directly observes that the behavior of Watson series is the opposite to that of canonical solution series. In particular, the number of terms increases with the distance *r*/*b* both from the source and the scattering surface; therefore, Watson transform determines more easily the wave function in the near field, where the variations are more abrupt than in the far field. In addition, the bigger the inclusion in terms of the wavelength gets, the less the required Watson terms are, unlike to what is happening with canonical terms.

In Fig. 4b, we investigate identically the scenarios of Fig. 1c and observe that the number of Watson terms to evaluate $\Psi _\textrm{int}$ at $r=g$ increases with *b*/*a*. In other words, even the most challenging for the canonical solutions case of $b\rightarrow r \rightarrow a$ is well treated by the Watson transform. Moreover, we note again, as in Fig. 4a, the decreasing trend of *U* with the optical radius $a/\lambda$ of the nanoinclusion which in the considered setup is quite substantial. Finally, the most important finding from Fig. 4 is that only a few (1–5) terms are enough for the evaluation of the respective wave functions. One may say that, practically, we have closed-form solutions; indeed, for the case that $a\gg \lambda$, which is the most common one, we have a single term defining the function $\Psi (r,\varphi )$ everywhere in the considered space. Therefore, the obtained result via Watson transform can be processed or optimized analytically; accordingly, they may shed light on effects regarding the spatial distribution of the probability $|\Psi |^2$ that are not easily understandable via the numerical evaluations of the canonical series.

**Fig. 5:**
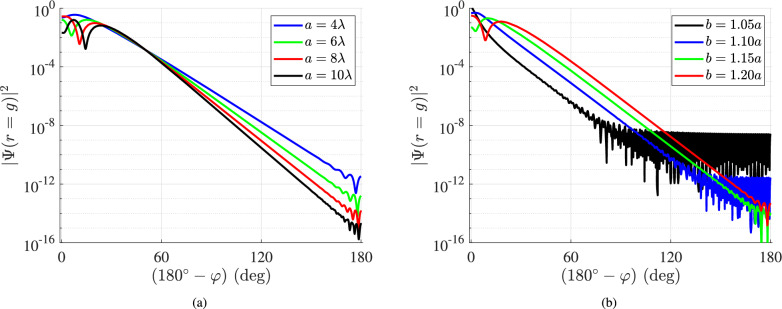
The probability $|\Psi |^2$ at $r=g\equiv (a+b)/2$ as a function of the complementary azimuthal angle $(180^{\circ }-\varphi )$: (**a**) for various optical sizes of the inclusion $a/\lambda$. ($b=1.2a$) and (**b**) for various relative locations of the source *b*/*a*. ($a=7\lambda$).

## Probability variations

It would be meaningful to use the Watson series (7) to compute the wave function $\Psi (r,\varphi )$ whose squared magnitude indicates the relative probability for finding a particle at a specific point $(r,\varphi )$. In Fig. 5a, we depict the variation of probability $|\Psi _\textrm{int}(r=g)|^2$, where $g=(a+b)/2$, with respect to the complementary azimuthal angle $(180^{\circ }-\varphi )$ for $0<\varphi <180^{\circ }$ because the system is symmetric for $-180^{\circ }<\varphi <0$. Since the structure is excited at $\varphi =180^{\circ }$, the probability is strong in the vicinity of the source and drops rapidly as the angle $(180^{\circ }-\varphi )$ increases and the observation point approaches the positive horizontal semi-axis. The decrease keeps up to the most distant point from the emitter and is more abrupt, the larger the scatterer gets.

In Fig. 5b, we repeat the same calculations but consider various locations of the source for a fixed $a=7\lambda$. Once again, the represented quantity declines as one moves towards the rear surface of the cylinder since the quantum interaction with the particle emitter becomes more and more indirect. Note that when the source is placed close to the inclusion, the probability $|\Psi |^2$ gets saturated and stabilized to specific small values; on the other hand, the drop is less rapid but continuous when one increases *b*/*a*. Finally, the variation of the probability near the source is more significant for larger *b*/*a* in the same way it was for more substantial $a/\lambda$ in Fig. 5a

**Fig. 6:**
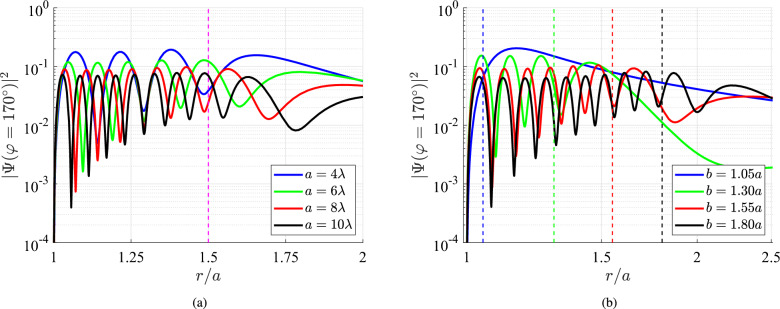
The probability $|\Psi |^2$ at $\varphi =\pm 170^{\circ }$ as a function of the normalized distance *r*/*a* from the surface: (**a**) for various sizes *a* in terms of $\lambda$ ($b=1.5a$), (**b**) for various locations of the source ($a=7\lambda$). The dashed lines indicate the radial positions of the source.

In Fig. 6a, we show the probability of a particle to exist along the directions $\varphi =\pm 170^{\circ }$ as a function of the normalized radial distance *r*/*a* for $b=1.5a$ while several optical radii of nanoinclusions are considered (the same as the ones of Fig. 5a). We observe oscillations in the region $a<r<b$ which get sharper with increasing *a*, simply because the considered distance is longer. Beyond the source location, which is indicated by a vertical dashed line, the quantity $|\Psi |^2$ fluctuates moderately and converges to specific values for $r\rightarrow +\infty$. It is also remarkable that the average values of the probability become more significant for smaller inclusions at $\varphi =\pm 170^{\circ }$ since the same overall $|\Psi |^2$ gets distributed around a scatterer with restricted circumference.

In Fig. 6b, we compute again the same quantity $|\Psi (\varphi =170^{\circ })|^2$ along the respective radius for various positions of the emitter *b*/*a*. Obviously, the probability vanishes at $r\rightarrow a$, as exactly happening in Fig. 6a, since the inclusion is impenetrable. Moreover, one can spot again that the number of resonances within the scatterer-source cavity $a<r<b$ are proportional to its size; indeed, if one places it slightly distant from the surface, the waves going back and forth do have the area to formulate oscillating patterns. Overall, the recorded behavior is similar to that of Fig. 6a, where the probability is (on average) increasing from $r=a$ to $r=b$ and mildly decreasing on the other side of the emitter.

**Fig. 7:**
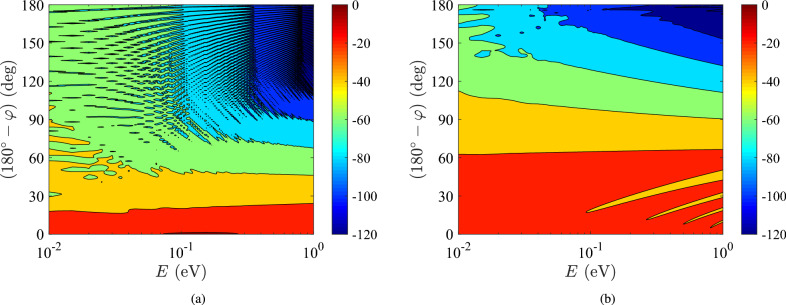
The probability $|\Psi |^2$ at $r=g\equiv (a+b)/2$ (in decibels) as a function of the impinging energy *E* (in eV) of matter waves and the complementary azimuthal angle $(180^{\circ }-\varphi )$ for: (**a**) $b=1.1a$, (**b**) $b=1.5a$. Inclusion size: $a=100~\textrm{nm}$, background: indium antimonide (InSb).

In Fig. 7, we consider a nanoinclusion with radius $a=100~\textrm{nm}$ that gets illuminated by quantum particles of a broad band of energies: $0.01~\textrm{eV}<E<1~\textrm{eV}$. The source is embedded into indium antimonide (InSb), whose effective electron mass reads^32,33^: $m_0=0.013m_e$ ($m_e$ is the inertial mass of electron into vacuum). In Fig. 7a, we locate the source close to the scatterer ($b=1.1a$) and notice that the probability $|\Psi _\textrm{int}(r=g)|^2$ drops more rapidly for high-energy particles as the observation point moves to $\varphi =0$ axis; indeed, matter waves of shorter wavelength travel a longer path around the inclusion and a tiny portion of their power reaches the rear side points. In Fig. 7b, the source gets more distant ($b=1.5a$) and we obtain same pattern appeared by Figs 5, 7a. However, the oscillations are less rapid compared to the aforementioned ones because the system is not fed at its very near field; as a result, the saturation effects of Fig. 5b are absent, unlike in Fig. 7a.

## Conclusions

Quantum scattering of beams with nanometer-sized wavelengths by either micrometer-sized inclusions into the atomic grid or impurities into the volume of condensed matter, has been studied. The canonical solutions employing partial wave formulation converge very slowly due to the huge optical diameter of the scatterers; therefore, Watson transform is implemented and an equivalent integral is obtained. It is evaluated via residue theorem with help from complex-order Hankel functions and an impressive improvement in the convergence behavior is observed. In most of the cases two or three residue terms are enough to compute reliably the wave function which is equivalent to giving closed-form solution to the problem; interestingly, the convergence of Watson series is faster once the scatterer gets optically bigger, while the canonical sum converges even more poorly. The equivalent series is used to determine the quantum fields both in the near and the far region as well as at the rear and the front side of the illuminated inclusion.

In this way, the Watson transform, even though it was initially introduced for electromagnetic waves, is demonstrated as a ubiquitous tool to solve efficiently the quantum interactions between impinging particles and objects within an arbitrary background semiconductor. Hence, an interesting expansion of the present work would be to consider impurities close to nanotips or other low-dimensional quantum structures^34,35^. In addition, the scatterers can be azimuthally non-entire^36^, symmetrically excited^37^, buried into media different from them where the primary source is positioned^38–40^ or placed along quantum metasurfaces^41^. Importantly, the inverse problem can be regarded where the scattering response can give textural or geometrical information about the object that perturbs the background quantum signal^42,43^. In all the aforementioned cases, classical electrodynamic setups can be utilized in applications within the quantum arena and Watson transform will render their analytical treatment feasible despite the ultrashort wavelengths of the incoming matter waves.

## Data Availability

The datasets used and/or analysed during the current study available from the corresponding author on reasonable request.
